# Severe Human Case of Zoonotic Infection with Swine-Origin Influenza A Virus, Denmark, 2021

**DOI:** 10.3201/eid2812.220935

**Published:** 2022-12

**Authors:** Klara M. Andersen, Lasse S. Vestergaard, Jakob N. Nissen, Sophie J. George, Pia Ryt-Hansen, Charlotte K. Hjulsager, Jesper S. Krog, Marianne N. Skov, Søren Alexandersen, Lars E. Larsen, Ramona Trebbien

**Affiliations:** Technical University of Denmark, Kongens Lyngby, Denmark (K.M. Andersen);; Statens Serum Institut, Copenhagen, Denmark (K.M. Andersen, L.S. Vestergaard, J.N. Nissen, C.K. Hjulsager, J.S. Krog, S. Alexandersen, R. Trebbien);; University of Copenhagen, Copenhagen (S.J. George, P. Ryt-Hansen, L.E. Larsen);; Odense University Hospital, Odense, Denmark (M.N. Skov)

**Keywords:** influenza A virus, viral zoonoses, Orthomyxoviridae Infections, influenza A virus, H1pdm09N1av subtype, Swine influenza virus, viruses, influenza, zoonoses, Denmark

## Abstract

During routine surveillance at the National Influenza Center, Denmark, we detected a zoonotic swine influenza A virus in a patient who became severely ill. We describe the clinical picture and the genetic characterization of this variant virus, which is distinct from another variant found previously in Denmark.

Human infections with swine influenza A viruses (IAVs) are sporadically reported (*1*–*4*). Increased surveillance has revealed substantial swine IAV circulation within pig herds and frequent reassortment with human seasonal IAVs (*5*). Despite no sustained human-to-human transmission of variant IAV cases since the 2009 influenza A(H1N1) pandemic, the zoonotic potential is of concern. We report a case of human infection with a swine-origin IAV that resulted in severe illness in a younger, otherwise healthy person employed at a swine slaughterhouse in Denmark. This case was detected 10 months after our previously reported case (*4*). The patient provided informed consent for publication of this case report.

On November 24, 2021, a person of ≈50 years of age was hospitalized after acute onset of illness characterized by dizziness on the night of November 23, 2021, followed by chest pain, pain radiating toward the left arm, diarrhea, and malaise that developed the next morning, but no fever. The patient called for emergency medical assistance, which arrived shortly. During ambulance transportation and at hospital arrival, the patient experienced repeated convulsions and was admitted to the intensive care unit and put on mechanical ventilation to manage seizures and associated reduced oxygen level. Extensive clinical examination, such as laboratory investigations (i.e., biochemical, microbiological, and immunological assays), multiorgan radiological examinations, and electroencephalography (Appendix 1), identified no cardiovascular, renal, neurologic, or other diseases that could explain the sudden severe illness. However, a tracheal sample collected and analyzed at the local microbiology laboratory was found positive for IAV (Appendix 1). No other microbiological agents were detected, including SARS-CoV-2 or other respiratory viruses, and the patient showed no signs of pneumonia. The patient received antiviral medication (oseltamivir) and various supportive treatments, and over the next 2 days the clinical condition improved; the patient was soon after discharged from the hospital.

The remaining sample material was submitted to the Danish National Influenza Center as part of routine influenza surveillance. The sample was confirmed positive for the pandemic H1N1 strain and was further analyzed by whole-genome sequencing (Appendix 1). Consensus sequences for the virus named A/Denmark/36/2021 were uploaded to GISAID (https://www.gisaid.org; isolate no. EPI_ISL_8786194). WGS confirmed the H1N1 subtype; however, the virus had closer similarity to swine IAVs (Figure) than to other human strains. BLAST (https://blast.ncbi.nlm.nih.gov/Blast.cgi) searches revealed no close matches to IAV sequences in GenBank or GISAID, but comparison to in-house sequences from the passive surveillance of influenza viruses in pigs from Denmark revealed close similarity to 2021 swine IAVs (Table). Phylogenetic analyses showed that most gene segments were related to the pandemic H1N1 subtype (clade 1A3.3.2), whereas the neuraminidase and nonstructural segments belonged to the clade 1C Eurasian avian-like swine influenza A(H1N1) (Figure; Appendix 1 Figures 1–7). In contrast, another variant virus found recently in Denmark had a clade 1C nonstructural segment, whereas the 7 other gene segments were related to clade 1A3.3.2 pandemic H1N1 viruses (*4*).

**Figure:**
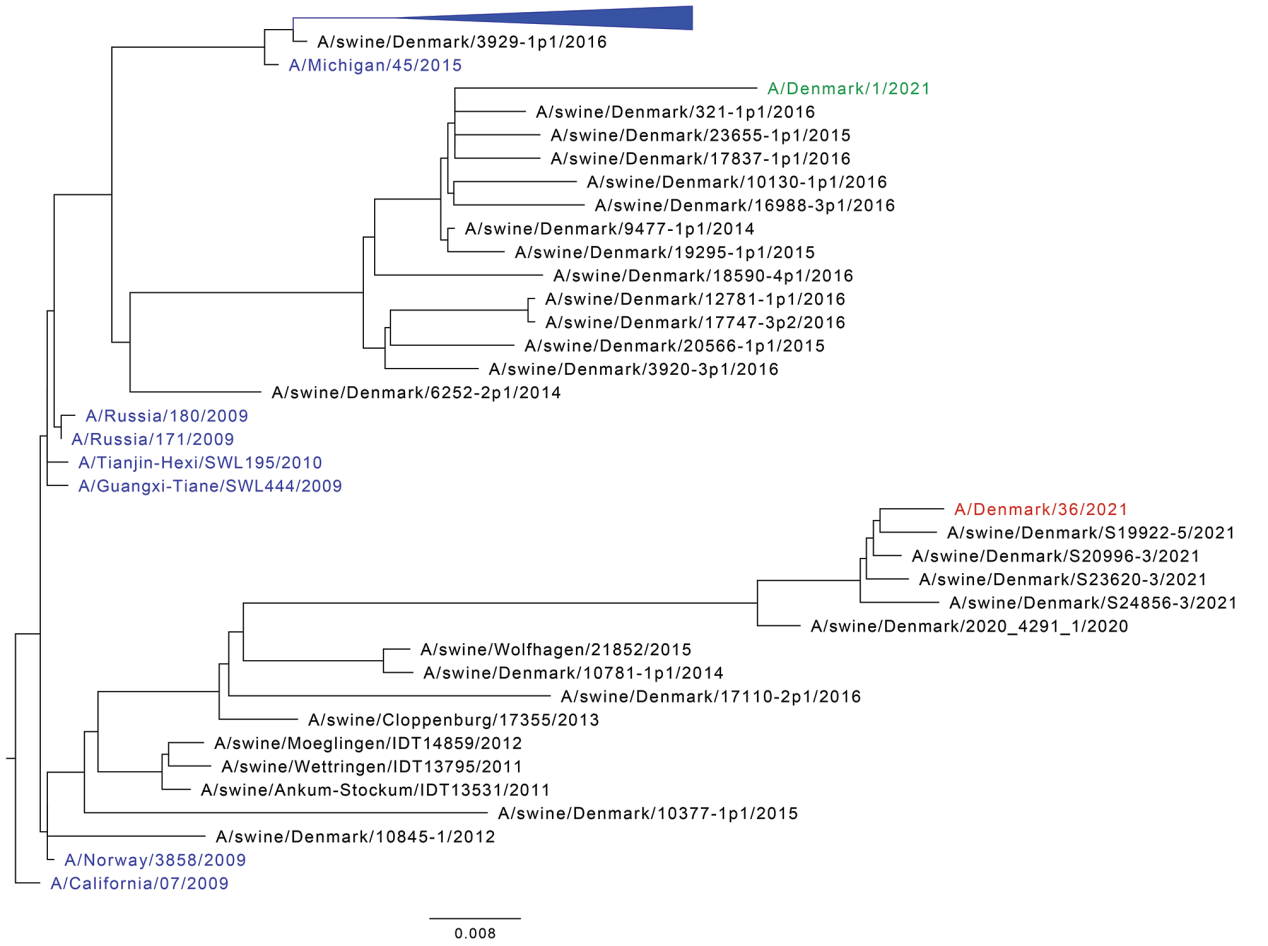
Maximum-likelihood phylogenetic tree of the hemagglutinin gene of influenza A virus from a patient in Denmark (A/Denmark/36/2021), the seasonal vaccine strain, and closely related strains. The tree includes the case variant virus A/Denmark/36/2021 (red), the 10 closest BLAST matches (https://blast.ncbi.nlm.nih.gov/Blast.cgi), the previously reported Denmark variant virus A/Denmark/1/2021 (green), human seasonal reference viruses with >85% nucleotide identity to A/Denmark/36/2021 (Appendix 2 Table), and representative viruses from the passive surveillance program of influenza viruses in pigs from Denmark. The tree is rooted on A/California/07/2009. Human IAV sequences are shown in blue, and most seasonal reference viruses have been collapsed. Scale bar indicates nucleotide substitutions per site.

**Table:** Percentage nucleotide and amino acid identities between influenza A virus from a patient in Denmark (A/Denmark/36/2021), the seasonal vaccine strain, and closely related strains*

Gene	A/Victoria/2570/2019	A/swine/Denmark/S19922-5/2021	A/swine/Denmark/24856-3/2021	A/swine/Denmark/S22282-5/2021	A/Denmark/1/2021
Pairwise nucleotide identity to A/Denmark/36/2021, %					
PB2	94.8	99.4	98.9	99.4	96.1
PB1	94.7	99.5	98.9	99.3	93.6
PA	95.2	99.8	99.3	99.6	96.8
HA	90.7	99.0	98.7	72.6	89.9
NP	95.3	99.7	98.9	99.4	96.4
NA	86.9	99.3	98.7	n/a	87.0
MP	95.2	99.6	99.6	92.6	95.3
NS	79.7	99.9	99.1	99.6	92.8
Pairwise amino acid identity to A/Denmark/36/2021, %					
PB2	97.2	99.6	99.5	99.6	97.9
PB1	97.6	99.9	99.5	99.9	97.9
PA	97.6	99.9	99.3	99.4	98.3
PA-X	96.6	100	100	100	98.7
HA	90.3	98.8	98.6	75.8	88.5
NP	98.2	100	99.6	99.8	98.4
NA	85.1	98.9	97.4	n/a	86.1
M1	97.6	100	100	97.6	97.6
M2	94.9	100	100	90.7	93.8
NS1	72.4	99.6	98.3	99.1	91.7
NEP	85.1	100	100	100	95

*Seasonal vaccine strain, A/Victoria/2570/2019 (GISAID isolate no. EPI_ISL_517733; Appendix 2; strains from passive surveillance Denmark: A/swine/Denmark/S19922–5/2021 (GenBank accession nos. ON716251–8), A/swine/Denmark/24856-3/2021 (accession nos. ON716275–82), A/Denmark/S22282-5/2021 (accession nos. ON716267–74); and another recent variant case found in Denmark (A/Denmark/1/2021; GISAID isolate no. EPI_ISL_909652) (*4*). The A/swine/Denmark/S22282-5/2021 is of the H1N2 subtype and therefore no percentage similarity is reported to the neuraminidase gene segment and protein of this strain. HA, hemagglutinin; MP/M1/M2, matrix protein 1/2; NA, neuraminidase; n/a, not applicable; NEP, nuclear export protein; NP, nucleoprotein; NS/NS1, nonstructural protein; PA, polymerase acidic protein; PB1/2, polymerase basic protein 1/2.

In-depth interviews with the patient revealed occupational exposure to swine in a pig slaughterhouse in Denmark, which appears the most likely place of infection. The patient handled live pigs, carcasses, and meat during the slaughtering process while wearing protective equipment including gloves and gown but no face mask. The patient was previously healthy, had no underlying diseases or immune deficiencies, and had received the recommended quadrivalent seasonal influenza vaccine in October 2021.

No other cases of influenza had been reported at the patient’s workplace or among close contacts. In the 2021–22 influenza season, 16,160 cases of influenza A virus occurred among 244,184 tested samples in Denmark; the H3N2 subtype was dominant. No other human cases of swine-origin influenza virus were detected during this period. Genetic analyses and antigenic characterization of the virus (Appendix 1 Table 1, Figure 8) showed several genetic and antigenic differences and suggested poor reactivity to the contemporary human seasonal influenza vaccine.

This reported case is considered independent of the previously reported variant infection in Denmark (*4*), because the 2 viruses are genetically distinct (Table). The symptoms were also different; the earlier case was in an elderly patient with comorbidities who experienced classical influenza-like illness, but in this case, a previously healthy adult of younger age experienced unusual severe and sudden illness. Influenza-associated convulsions in adults are rare (*6*) and mostly accompanied by fever or encephalitis, which was not observed in this patient.

The identification of variant IAVs emphasizes the zoonotic potential of these strains and highlights the importance of continued monitoring of both human and swine IAVs. The reported case suggests a need for focusing on early registration of swine exposure for humans with influenza-like illness, as well as increased measures to reduce the swine IAV exposure risk for people with occupational contact with swine.

Appendix 1 Additional information about severe human case of zoonotic infection with swine-origin influenza a virus A/H1pdm09N1av-like, Denmark, 2021

Appendix 2 Additional data used in study of severe human case of zoonotic infection with swine-origin influenza a virus A/H1pdm09N1av-like, Denmark, 2021
