# Supplementary material for: Severe Human Case of Zoonotic Infection with Swine-Origin Influenza A Virus, Denmark, 2021
Source: Emerg Infect Dis. 2022 Dec;28(12):2561–4. doi: 10.3201/eid2812.220935 (PMC9707568; doi:10.3201/eid2812.220935)
Supplement: Appendix 1 — Additional information about severe human case of zoonotic infection with swine-origin influenza a virus A/H1pdm09N1av-like, Denmark, 2021 [file 22-0935-Techapp-s1.pdf]

Article DOI: https://doi.org/10.3201/eid2812.220935

Severe Human Case of Zoonotic Infection with Swine-Origin Influenza A Virus, Denmark, 2021

Appendix

Methods for Virus Detection and Analysis

A tracheal sample was collected and analyzed at the local hospital microbiology laboratory using Cepheid Xpert Xpress SARS-CoV-2_Flu_RSV assay (Cepheid, Sunnyvale, CA). The sample had a cp-value of 23.9 for assay target FluA 1 and a cp-value of 26.3 for the assay target FluA 2. The sample was negative for Influenza B, SARS-CoV-2 and RSV.

At the National Influenza Center at Statens Serum Institut, Copenhagen, Denmark, the remaining sample material was analyzed by in-house real-time RT-PCR which detects the matrix- and H1pdm09-gene segments. Here, the sample was confirmed influenza A H1pdm09 positive with Ct values of 21.36 for the matrix gene and 23.41 for the H1pdm09 gene.

In addition, the sample was amplified by one-tube RT-PCR (*1*) and sequenced on the MiSeq platform (Illumina) using the Nextera XT DNA library preparation kit (Illumina) following the manufacturer's instructions.

Consensus sequences for each segment were made using an in-house pipeline. Briefly, raw sequencing reads were quality trimmed using fastp and consensus sequences for each segment were made using an iterative mapping approach with KMA. In a first step, raw reads were mapped against a large reference database with KMA, and the top few reference sequences for each segment were picked for making a reference-based assembly. The reads were then aligned again to this first assembly, and if the two assemblies were identical, this would be outputted as the consensus sequence. If not, the reads would again be aligned to the second assembly and this step would be repeated until the assemblies converged or until a maximum number of iterations.

For phylogenetic analysis, the consensus sequences were aligned to human reference sequences, other swine IAV sequences etc. with MAFFT, alignments were trimmed with trimAl (-gt 0.9 -cons 60) and maximum likelihood phylogenetic trees were built with IQ-TREE using the HKY+G2 method.

Genetic and Antigenic Characterization

Virus isolation was unsuccessful, but antigenic characterization by hemagglutinin inhibition (HAI) test was performed on a culture of the closely related swIAV, A/swine/Denmark/19922–5/2021 (Figure [https://wwwnc.cdc.gov/EID/article/28/12/22-0935-F1.htm]; Appendix 1 Figure 8), which showed poor cross-reactivity to all used reference antisera (Appendix 2, https://wwwnc.cdc.gov/EID/article/28/12/22-0935-App2.xlsx). Also, analysis of the case virus genome sequences showed that it was distinct in all genes (79.7% – 95.3% nucleotide identity) from the contemporary human seasonal influenza vaccine virus A/Victoria/2570/2019 (Table) and contained several differences in the antigenic sites of the Hemagglutinin (HA) protein (Appendix 1 Figure 8). Scanning through protein sequences of all segments, we identified a few amino acid substitutions previously reported to be involved in host specificity, increased pathogenicity or polymerase activity (PB2: T588I; PB1: G216S, Q584H; NP: I100M) (*4–7*). The NA protein contained no substitutions known to confer antiviral resistance.

Clinical, Laboratory and Other Examinations Performed to Understand the Severe Course of Illness

A wide range of clinical, laboratory and scanning examinations were performed to reveal any other underlying illness to explain the severe clinical condition of the reported swine flu case.

Microbiological Testing

Microbiological testing included the following patient specimens: Blood culture, cerebrospinal fluid (CSF), tracheal swap, urine sample, fecal sample. The following organisms were tested for using a variety of methods, including culture, microscopy and PCR.

CSF: No observed mononuclear nor neutrophil leukocytes. No observed bacterial nor fungal growth. *Escherichia coli* K1 negative. Haemophilus influenza negative. Listeria monocytogenes negative. Neisseria meningitides negative. Group B streptococcus negative.

Streptococcus pneumonia negative. Epstein-Barr virus negative. Herpes Simplex virus type 1 and 2 negative. Influenza virus A and B negative. Enterovirus negative Cytomegalovirus negative. Human herpesvirus 6 negative. Human parechovirus negative. Varicella zoster virus negative. Cryptococcus neoformans/gattii negative.

Tracheal swap: No fungal growth. **Influenza virus A positive**. Influenza virus B negative. Coronavirus SARS-CoV-2 negative.

Urine: No bacterial or fungal growth.

Blood and CSF Biochemistry

Leukocytes elevated 14.7 × 10E9/L (normal range 3.50–8.80), Erythrocytes normal 0.41 (normal range 0.35–0.46), Hemoglobin 8.3 mmol/L (normal range 7.3–9.5), thrombocytes 216 × 10E9/L (normal range 165–400). Coagulation factors and liver enzymes all within normal ranges.

C-reactive protein (CRP) showed normal levels at time of admission, but was elevated on day 2 at 76 mg/L.

Immunological and inflammatory markers all within normal ranges. Autoimmune encephalitis markers all within normal ranges.

Radiological Examinations

Radiological examinations including chest x-ray, abdominal x-ray, chest CT scan, angiography CT scan, brain CT and MR scan were performed at the time of admission and repeated during hospitalization, which all showed normal conditions and no pathology.

Electroencephalography

To further understand the possible cause of observed convulsions, electroencephalography was performed 2 months after the illness episode, which showed normal conditions.

References:

1. Zhou B, Donnelly ME, Scholes DT, St George K, Hatta M, Kawaoka Y, et al. Single-reaction genomic amplification accelerates sequencing and vaccine production for classical and Swine origin

human influenza a viruses. J Virol. 2009;83:10309–13. PubMed https://doi.org/10.1128/JVI.01109-09

2. Brownlee GG, Fodor Sir William E. The predicted antigenicity of the haemagglutinin of the 1918 Spanish influenza pandemic suggests an avian origin. Philos Trans R Soc Lond B Biol Sci. 2001;356:1871–6. **PMID 11779386**

3. Burke DF, Smith DJ. A recommended numbering scheme for influenza A HA subtypes. PLoS One. 2014;9:e112302 PubMed https://doi.org/10.1371/journal.pone.0112302

4. Zhao Z, Yi C, Zhao L, Wang S, Zhou L, Hu Y, et al. PB2-588I enhances 2009 H1N1 pandemic influenza virus virulence by increasing viral replication and exacerbating PB2 inhibition of beta interferon expression. J Virol. 2014;88:2260–7. PubMed https://doi.org/10.1128/JVI.03024-13

5. Lin RW, Chen GW, Sung HH, Lin RJ, Yen LC, Tseng YL, et al. Naturally occurring mutations in PB1 affect influenza A virus replication fidelity, virulence, and adaptability. J Biomed Sci. 2019;26:55. PubMed https://doi.org/10.1186/s12929-019-0547-4

6. Gíria M, Rebelo de Andrade H. Genetic evolution of PB1 in the zoonotic transmission of influenza A(H1) virus. Infect Genet Evol. 2014;27:234–43. PubMed https://doi.org/10.1016/j.meegid.2014.07.024

7. Dornfeld D, Petric PP, Hassan E, Zell R, Schwemmle M. Eurasian avian-like swine influenza A viruses escape human MxA restriction through distinct mutations in their nucleoprotein. J Virol. 2019;93:e00997-18. PubMed https://doi.org/10.1128/JVI.00997-18

Appendix 1 Table. Results from a Hemagglutination Inhibition test of the closely related swine IAV, A/swine/Denmark/S19922–5/2021 tested against reference ferret antisera of A/H1N1 viruses provided by WHO CC, Francis Crick Institute, UK. Average titer values from duplicate tests of cross-reactivity between the indicated viruses and antisera are indicated. A titer <20 was considered no reaction.

Reference viruses:	A/Victoria/2570/2019	A/Wisconsin/588/2019	A/Denmark/3280/2019	A/Guangdong-Maonan/swl1536/2019	A/California/07/09	A/Michigan/45/2015	A/Brisbane/02/2018
A/Victoria/2570/19	640						
A/Wisconsin/588/2019		1280					
A/Denmark/3280/2019			>2560				
A/Guangdong-Maonan/swl1536/2019				2560			
A/California/07/09					640		
A/Michigan/45/2015						640	
A/Brisbane/02/2018							1280
Sample virus: A/swine/Denmark/S19922–5/2021	<20	<20	<20	<20	<20	<20	<20

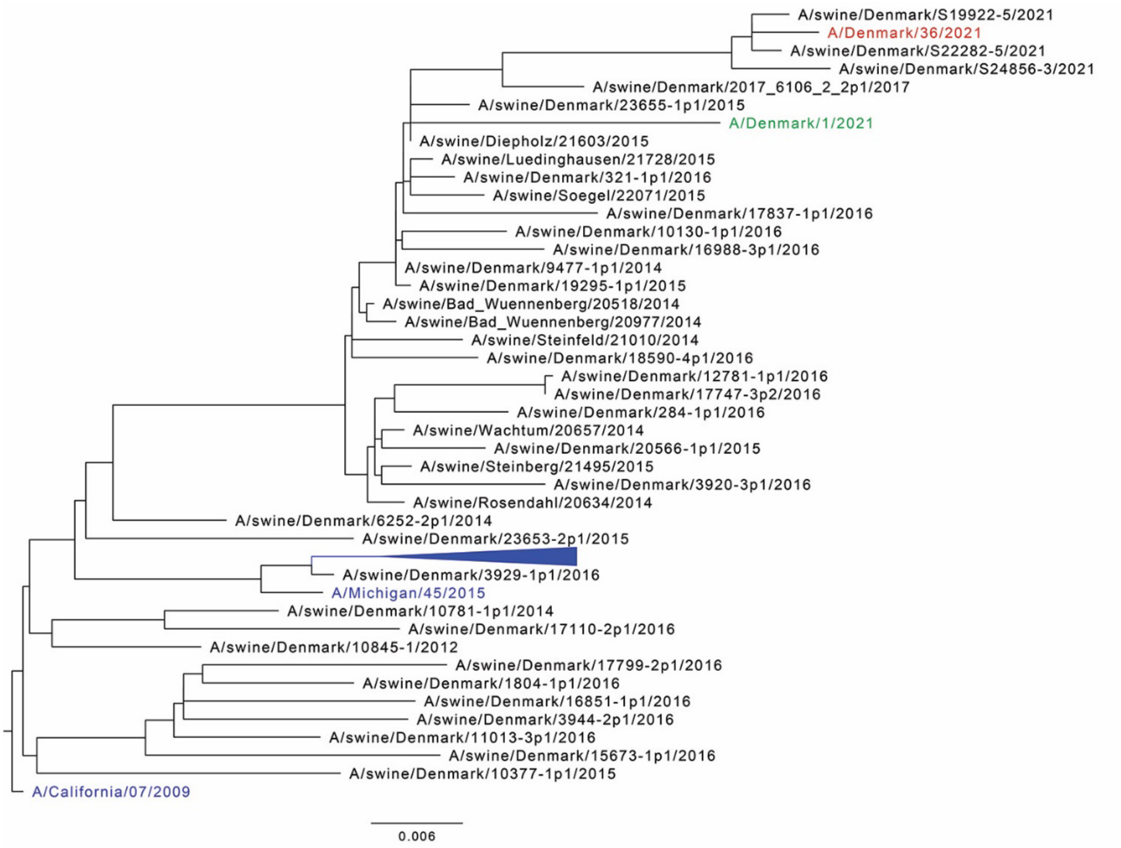

Appendix 1 Figure 1. Maximum-likelihood phylogenetic tree of the Polymerase Basic 2 (PB2) gene segment. The tree includes the case variant virus A/Denmark/36/2021 (red), the 10 closest BLAST matches (https://blast.ncbi.nlm.nih.gov/Blast.cgi), the previously reported Danish variant virus A/Denmark/1/2021 (green), human seasonal reference viruses with >85% nucleotide identity (Appendix 2, https://wwwnc.cdc.gov/EID/article/28/12/22-0935-App2.xlsx), and representative viruses from the passive surveillance program of influenza viruses in Danish pigs. The tree is rooted on A/California/07/2009. Human influenza A virus sequences are shown in blue, and most seasonal reference viruses have been collapsed. The scale bar indicates nucleotide substitutions per site.

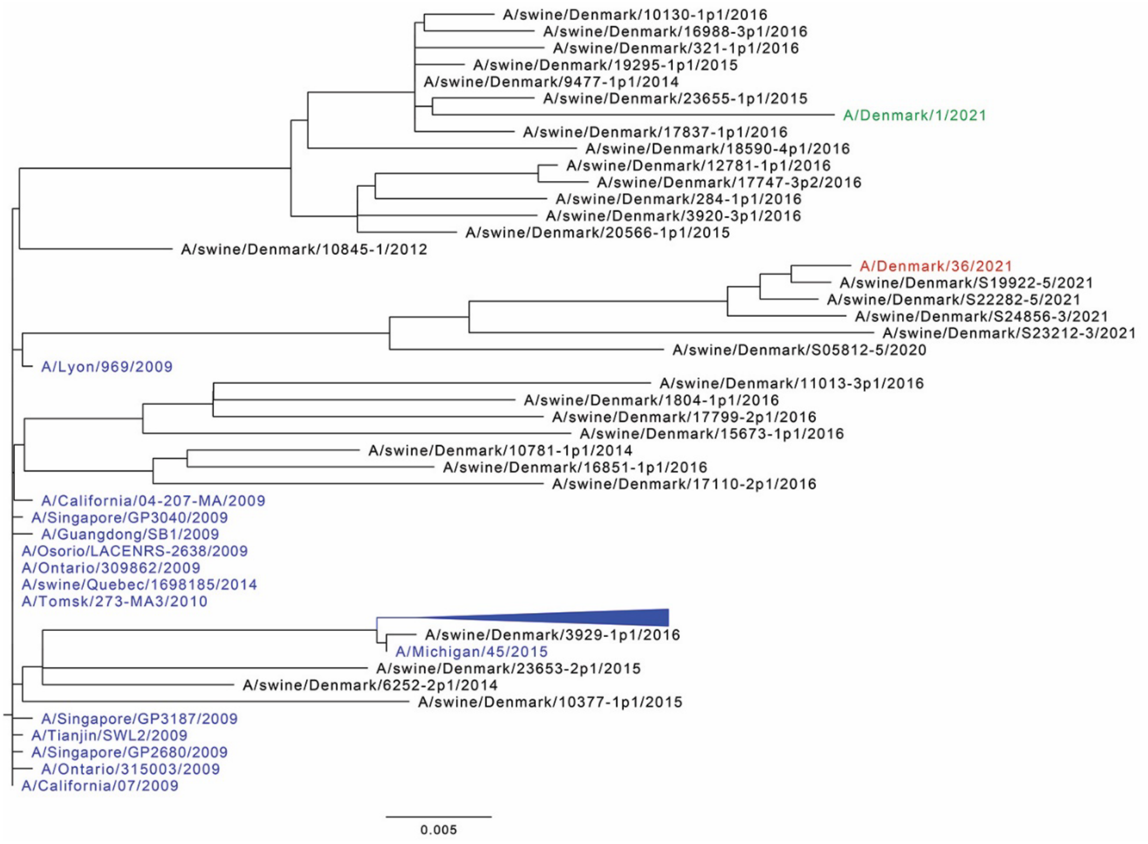

Appendix 1 Figure 2. Maximum-likelihood phylogenetic tree of the Polymerase Basic 1 (PB1) gene segment. The tree includes the case variant virus A/Denmark/36/2021 (red), the 10 closest BLAST matches (https://blast.ncbi.nlm.nih.gov/Blast.cgi), the previously reported Danish variant virus A/Denmark/1/2021 (green), human seasonal reference viruses with >85% nucleotide identity (Appendix 2, https://wwwnc.cdc.gov/EID/article/28/12/22-0935-App2.xlsx), and representative viruses from the passive surveillance program of influenza viruses in Danish pigs. The tree is rooted on A/California/07/2009. Human influenza A virus sequences are shown in blue, and most seasonal reference viruses have been collapsed. The scale bar indicates nucleotide substitutions per site.

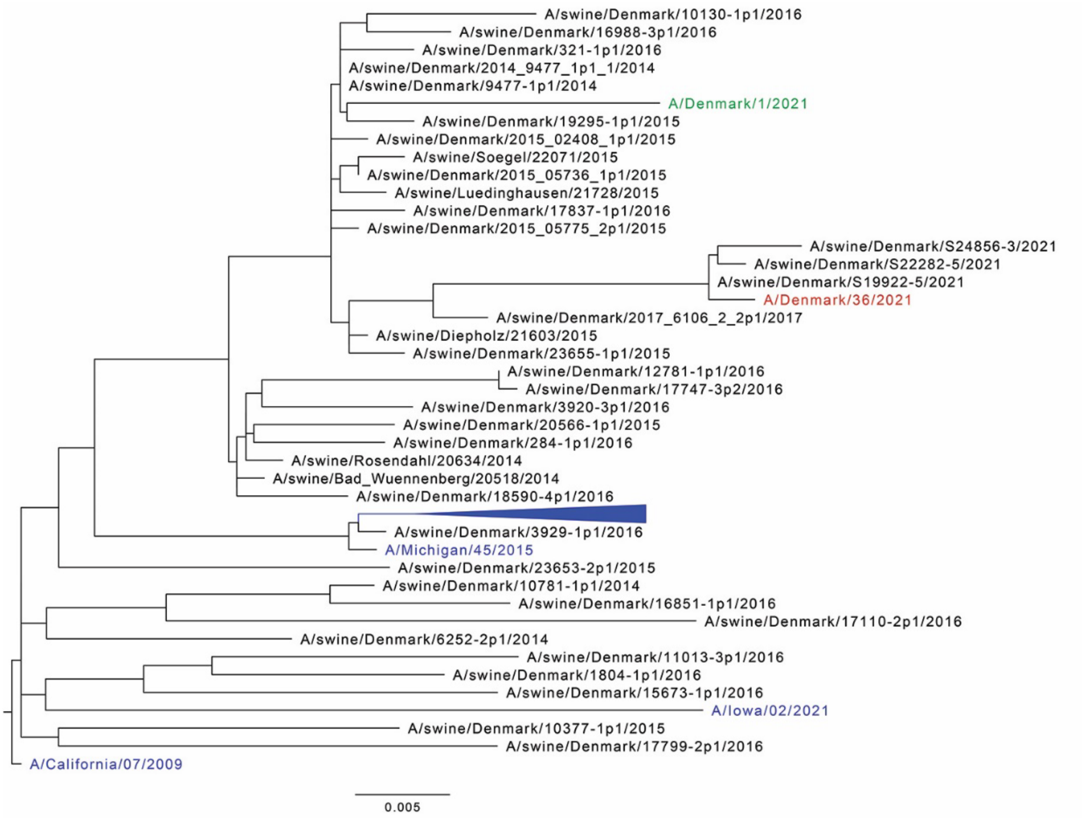

Appendix 1 Figure 3. Maximum-likelihood phylogenetic tree of the Polymerase Acidic (PA) gene segment. The tree includes the case variant virus A/Denmark/36/2021 (red), the 10 closest BLAST matches (https://blast.ncbi.nlm.nih.gov/Blast.cgi), the previously reported Danish variant virus A/Denmark/1/2021 (green), human seasonal reference viruses with >85% nucleotide identity (Appendix 2, https://wwwnc.cdc.gov/EID/article/28/12/22-0935-App2.xlsx), and representative viruses from the passive surveillance program of influenza viruses in Danish pigs. The tree is rooted on A/California/07/2009. Human influenza A virus sequences are shown in blue, and most seasonal reference viruses have been collapsed. The scale bar indicates nucleotide substitutions per site.

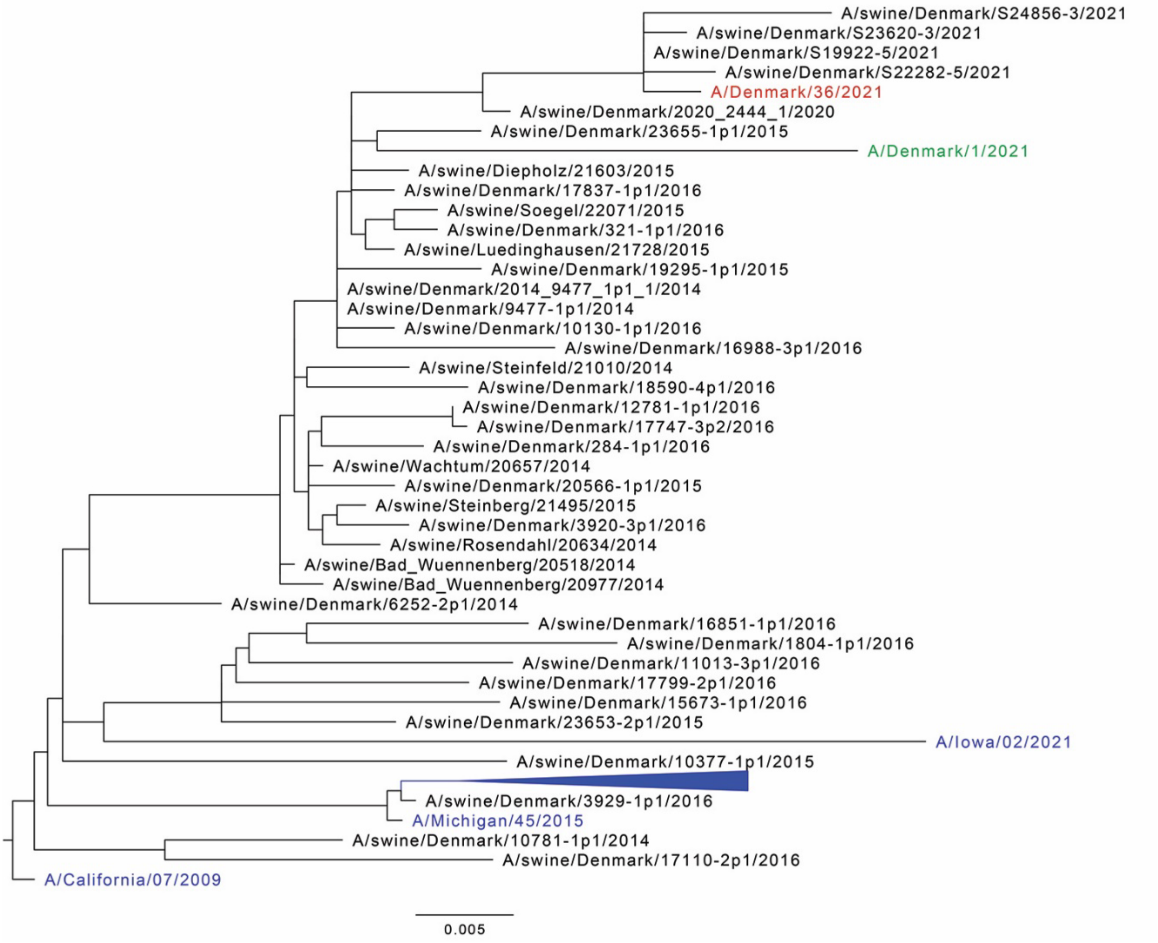

Appendix 1 Figure 4. Maximum-likelihood phylogenetic tree of the Nucleoprotein (NP) gene segment. The tree includes the case variant virus A/Denmark/36/2021 (red), the 10 closest BLAST matches (https://blast.ncbi.nlm.nih.gov/Blast.cgi), the previously reported Danish variant virus A/Denmark/1/2021 (green), human seasonal reference viruses with >85% nucleotide identity (Appendix 2, https://wwwnc.cdc.gov/EID/article/28/12/22-0935-App2.xlsx), and representative viruses from the passive surveillance program of influenza viruses in Danish pigs. The tree is rooted on A/California/07/2009. Human influenza A virus sequences are shown in blue, and most seasonal reference viruses have been collapsed. The scale bar indicates nucleotide substitutions per site.

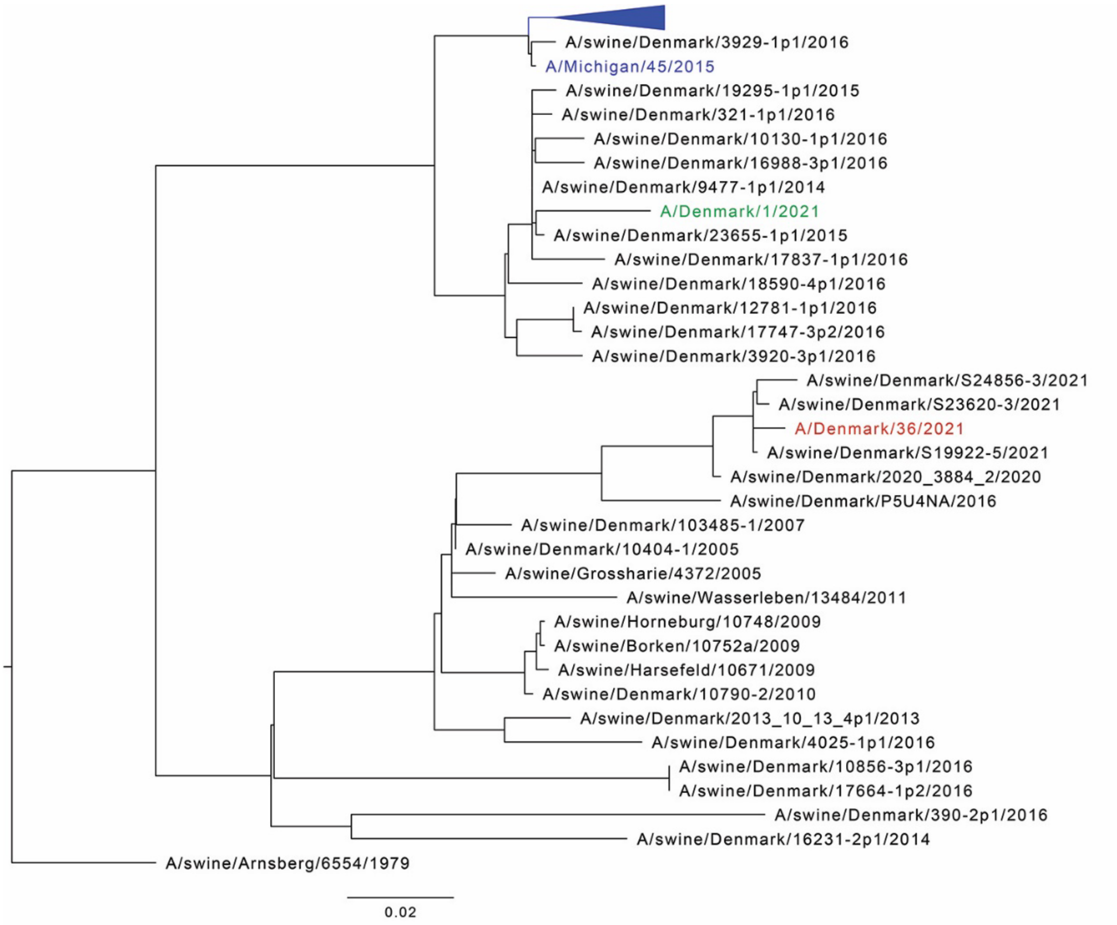

Appendix 1 Figure 5. Maximum likelihood phylogenetic tree of the Neuraminidase (NA) gene segment. The tree includes the case variant virus A/Denmark/36/2021 (red), the ten closest BLAST matches (https://blast.ncbi.nlm.nih.gov/Blast.cgi), the previously reported Danish variant virus A/Denmark/1/2021 (green), human seasonal reference viruses with >85% nucleotide identity (Appendix 2, https://wwwnc.cdc.gov/EID/article/28/12/22-0935-App2.xlsx), and representative viruses from the passive surveillance program of influenza viruses in Danish pigs. The tree is rooted on A/swine/Arnsberg/6554/1979. Human influenza A virus sequences are shown in blue, and most seasonal reference viruses have been collapsed. The scale bar indicates nucleotide substitutions per site.

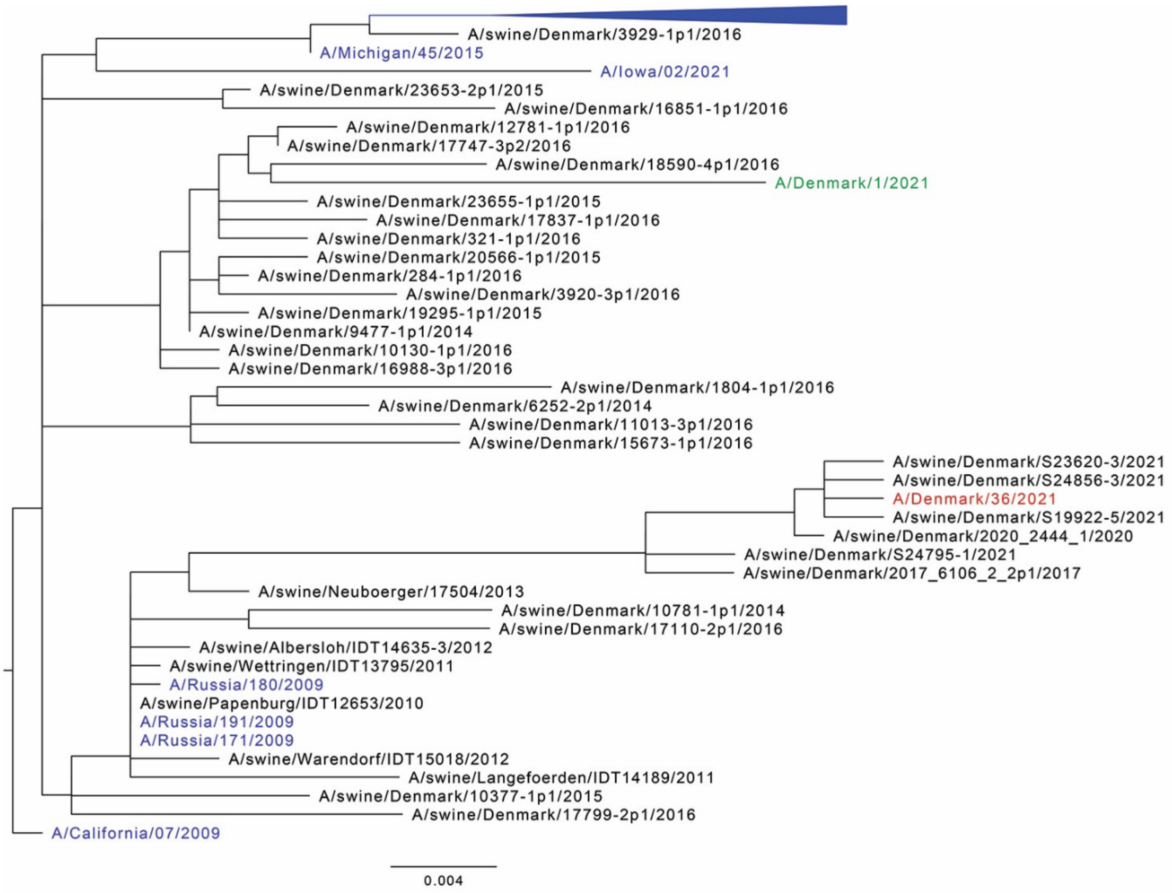

Appendix 1 Figure 6. Maximum likelihood phylogenetic tree of the Matrix Protein (MP) gene segment. The tree includes the case variant virus A/Denmark/36/2021 (red), the ten closest BLAST matches (https://blast.ncbi.nlm.nih.gov/Blast.cgi), the previously reported Danish variant virus A/Denmark/1/2021 (green), human seasonal reference viruses with >85% nucleotide identity (Appendix 2, https://wwwnc.cdc.gov/EID/article/28/12/22-0935-App2.xlsx), and representative viruses from the passive surveillance program of influenza viruses in Danish pigs. The tree is rooted on A/California/07/2009. Human influenza A virus sequences are shown in blue, and most seasonal reference viruses have been collapsed. The scale bar indicates nucleotide substitutions per site.

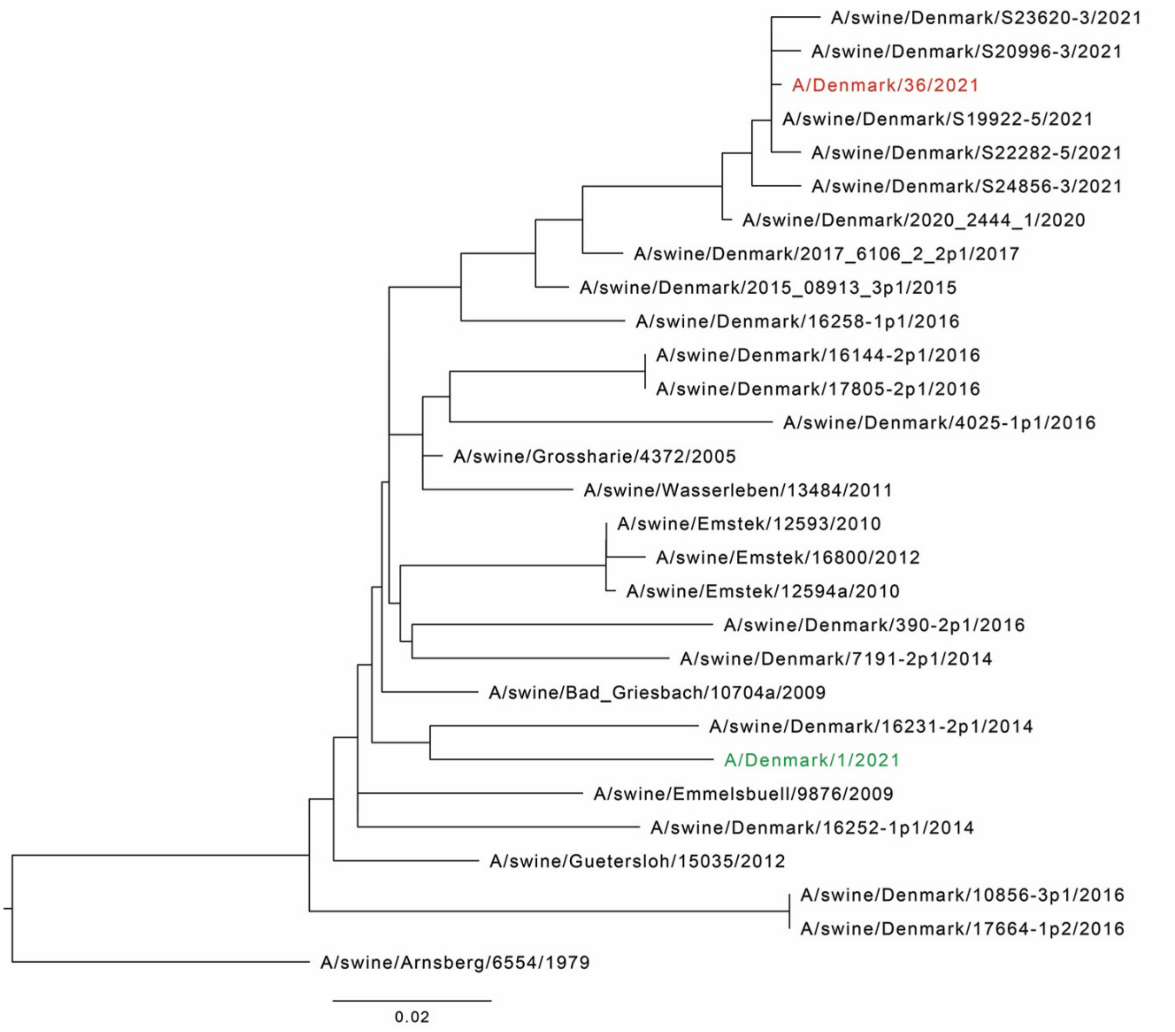

Appendix 1 Figure 7. Maximum likelihood phylogenetic tree of the Nonstructural (NS) gene. The tree includes the case variant virus A/Denmark/36/2021 (red), the ten closest BLAST matches (https://blast.ncbi.nlm.nih.gov/Blast.cgi), the previously reported Danish variant virus A/Denmark/1/2021 (green), and representative viruses from the passive surveillance program of influenza viruses in Danish pigs. The tree is rooted on A/swine/Arnsberg/6554/1979. No human seasonal reference viruses had >85% nucleotide identity to this segment. The scale bar indicates nucleotide substitutions per site.

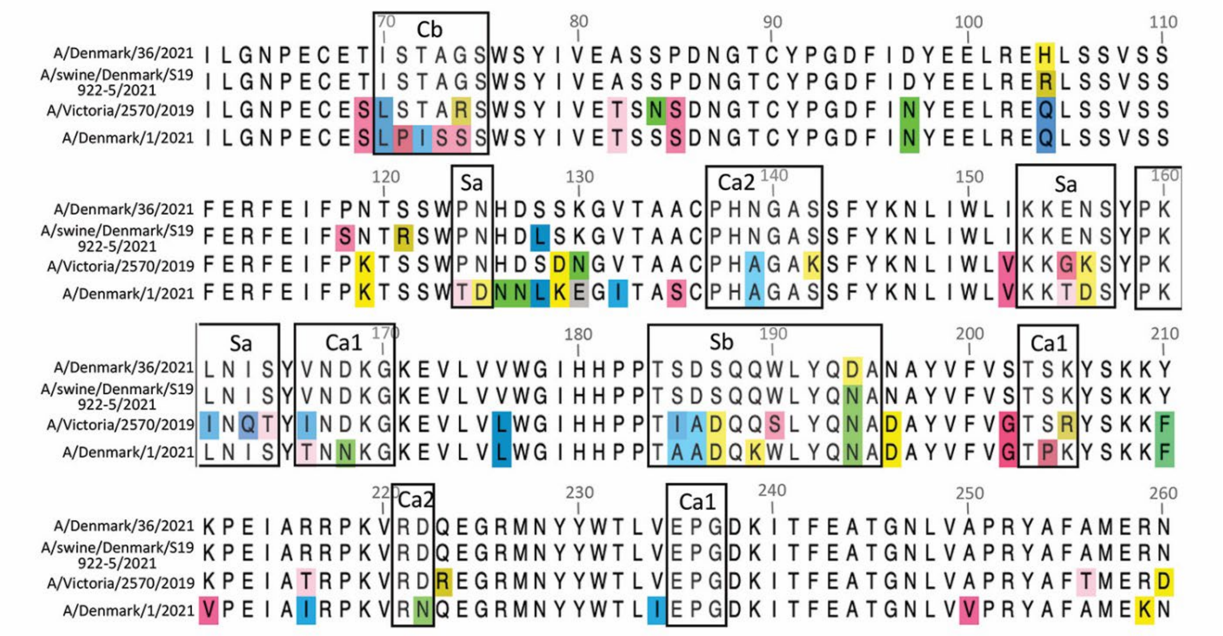

Appendix 1 Figure 8. Alignment of the HA amino acid sequences of the case variant virus A/Denmark/36/2021, the closely related swine influenza virus A/swine/Denmark/S19922–5/2021, the seasonal vaccine strain A/Victoria/2570/2019 (Appendix 2, https://wwwnc.cdc.gov/EID/article/28/12/22-0935-App2.xlsx), and the previously reported Danish variant virus A/Denmark/1/2021. Mutations relative to each other are highlighted in colors, and framed boxes show antigenic sites as defined by Brownlee and Fodor (*2*). Only part of the total HA alignment is shown with H1 numbering starting after the signal peptide (*3*).
